# Are Cardiovascular Risk Factors Associated with Verbal Learning and Memory Impairment in Patients with Schizophrenia? A Cross-Sectional Study

**DOI:** 10.1155/2012/204043

**Published:** 2012-11-19

**Authors:** Christophe Lancon, Daniel Dassa, Jessica Fernandez, Raphaelle Richieri, Romain Padovani, Laurent Boyer

**Affiliations:** ^1^EA 3279—Public Health, Chronic Disease, and Quality of Life Research Unit, Aix-Marseille University, 13005 Marseille, France; ^2^Department of Psychiatry, Sainte-Marguerite University Hospital, 13009 Marseille, France; ^3^Department of Addiction, Sainte-Marguerite University Hospital, 13009 Marseille, France; ^4^Department of Psychiatry, La Conception University Hospital, 13009 Marseille, France

## Abstract

*Objective*. The aim of this study is to assess the relationships of cardiovascular risk factors with verbal learning and memory in patients with schizophrenia. *Methods and Design*. cross-sectional study. *Inclusion Criteria*. Diagnosis of schizophrenia according to the DSM-IV-TR criteria. *Data Collection*. Sociodemographic information, clinical characteristics, anthropometric measurements, blood tests, and episodic memory using the California Verbal Learning Test (CVLT). *Analysis*. A multivariate analysis using multiple linear regressions was performed to determine variables that are potentially associated with verbal learning and memory. *Results*. One hundred and sixty-eight outpatients participated in our study. An association was found between the metabolic syndrome (MetS) and memory impairment on measures of verbal learning, and short- and long-term memory. Among the different components of MeTS, hypertriglycerides, abdominal obesity, and low HDL cholesterol were the only factors associated with memory impairment. Alcohol dependence or abuse was associated with a higher rate of forgetting. *Conclusion*. Our findings suggest that MetS and alcohol use may be linked with memory impairment in schizophrenia. These findings provide important insights into the interdependencies of cardiovascular risk factors and cognitive disorders and support novel strategies for treating and preventing cognitive disorders in patients with schizophrenia.

## 1. Introduction

Schizophrenia is characterized by significant abnormalities in multiple neurocognitive processes [1]. Cognitive impairment, especially in verbal learning and memory [2], is considered to be a primary reason for functional disability even after successful treatment and reduction of psychotic symptoms [3]. This area of investigation demands thus attention. Identification of potentially modifiable determinants of verbal learning and memory in schizophrenia is of importance for developing effective interventions that can improve the cognitive performance of patients. Interestingly, evidence suggests that cardiovascular risk factors affect cognitive functions in older patients [4–7]. Moreover, the prevalence of metabolic syndrome (MetS) is higher in individuals with schizophrenia than in the general population, with an overall rate ranging from 30 to 35% [8]. Atypical or second-generation antipsychotics are especially associated with obesity and other components of metabolic syndrome, particularly abnormal glucose and lipid metabolism [9]. Individuals with a psychiatric disorder consume approximately 46% of all cigarettes smoked in the United States [10]. Recent studies also suggest that 20–50% of schizophrenia patients have a comorbid alcohol use disorder [11]. Unfortunately, data are scarce on the links between cardiovascular risk factors and verbal learning and memory in schizophrenia [12, 13]. The most recent work suggested that hypertension and a body mass index >25 were associated to memory impairment [13]. Therefore, in the present study, our aim was to investigate the relationships of MetS, alcohol use disorder, and tobacco use with verbal learning and memory in patients with schizophrenia.

## 2. Methods

### 2.1. Study Participants

The study evaluated all prospective patients attending daytime hospital hours in our university and psychiatric hospital (Sainte-Marguerite University Hospital, Marseille, France) over a period of 12 months, from January 2011 to December 2011. All patients provided written informed consent. The inclusion criteria were the following: being over 18 years of age, having a diagnosis of schizophrenia according to the Diagnostic and Statistical Manual of Mental Disorders, 4th ed. (DSM-IV-TR) criteria [14], and speaking French as one's native language. The exclusion criteria included the following: reduced capacity to consent, diagnosis other than schizophrenia on Axis I of the DSM-IV, decompensated organic disease (i.e., appearance or exacerbation of organic disease which does not allow the patient to have a clinical interview), mental retardation (Intelligence Quotient <70), and primary neurologic conditions such as traumatic brain injury, cerebrovascular accident, or transient ischemic attack. All clinical assessments are performed in routine practice in our university psychiatric center. According to the Article L1121-1, Loi n°2011-2012 du 29 décembre 2011 - art. 5, ethical approval is not needed in France for researches in which all actions are performed and products used routinely. This study was conducted in accordance with the Declaration of Helsinki and French Good Clinical Practices. 

### 2.2. Data Collection

Clinical assessments were performed by a multidisciplinary team including a psychiatrist, a clinical psychologist, a nurse, and a social worker if appropriate. The following data were collected.

1. Sociodemographic information: age, gender, and educational level.
2. Clinical characteristics: duration of disease; psychotic symptoms based on the Positive and Negative Syndrome Scale (PANSS), which comprises three different subscales such as positive, negative and general psychopathology [15]; smoking status assessed by the Fagerstrom Test for Nicotine Dependence (FTND) [16]; and alcohol dependence or abuse determined by the DSM-IV-TR (during the past year) [14].
3. Drug information: antipsychotic medications (first-generation antipsychotics (FGAs), second-generation antipsychotics (SGAs)).
4. Memory assessment: all participants completed the California Verbal Learning Test (CVLT). Administration procedures for the CVLT [17] are as follows: List A, a 16-word list, is presented five consecutive times (Trials 1–5). This list is comprised of words equally divided among four semantic categories (spices and herbs, clothing, tools, and fruits). Word order is consistent over each trial and is not related to semantic category membership. At the end of each presentation, the subject is instructed to repeat as many words as possible, in any order. After completion of Trial 5, a second 16-item List, List B, is presented once. List B is comprised of words in similar (i.e., spices and herbs, fruits) and different (i.e., fish, cooking utensils) semantic categories. After presentation of List B, the subject is asked to repeat as many words as possible from that list, in any order. After recall of List B, the subject is asked to repeat as many words as possible from List A in a short-delay free recall trial. Next, in the short-delay cued recall trial, the subject is provided with descriptors, or semantic cues, for each of the four categories of List A words (e.g., “Tell me all the words on the list that are tools.”) to help the subject recall words not initially remembered. After approximately 20 min, the subject is asked to repeat List A, both in free and cued long-delay recall conditions. The same senior psychologist, who was trained intensively in test administration and was not involved in the treatment of the individuals, administered the tests in a standardised manner. The same instructions were given to the individuals prior to each trial.
5. Metabolic syndrome: diagnosis of MetS was defined according to the National Cholesterol Education Program's ATP-III criteria [18], including 3 or more of the following criteria: waist circumference >88 cm in women and >102 cm in men, fasting serum triglycerides ≥150 mg/dL; serum HDL <50 mg/dL in women and <40 mg/dL in men, blood pressure ≥130/85 mmHg (or treatment of previously diagnosed hypertension) and fasting blood glucose levels ≥110 mg/dL. Waist circumference was measured at the midpoint between the lower rib margins and the iliac crest. Arterial blood pressure was recorded using a standard mercury sphygmomanometer. Glucose and lipoprotein concentrations were analysed in fasting venous blood samples using standard enzymatic techniques.

### 2.3. Statistical Analyses 

Data were expressed as proportions, means or standard deviations. We performed unadjusted and multivariate-adjusted linear regression analyses, including age, sex, education level, MetS, smoking, and alcohol usage to determine variables potentially associated with memory impairments on measures of span of apprehension (List A, trial 1), verbal learning (total recall trials 1–5, List A), short-term memory (short delay free recall, short delay cued recall), long-term memory (long delay free recall, long delay cued recall), rate of forgetting (difference between short and long-delay free recall), and interference (proactive: difference between List B and List A, trial 1; retroactive: difference between short delay free recall and List A, trial 5). The analyses were repeated using MetS as a continuous variable (number of the MetS criteria present) according to Friedman et al. [13], and for each component of MetS (waist circumference, triglycerides, HDL cholesterol levels, blood pressure, and glycaemia). All the tests were two-sided. Statistical significance was defined as *P* < 0.05. The statistical analyses were performed using the SPSS version 18.0 software package (SPSS Inc., Chicago, IL, USA).

## 3. Results

One hundred and sixty-eight outpatients with schizophrenia participated in our study. The characteristics of the patients are listed in Table 1. The MetS prevalence rate among the 168 patients was 27.4%. The prevalence rate for individual components of MeTS was 46.4% for abdominal obesity, 22.6% for high triglycerides, 35.7% for low HDL cholesterol, 39.9% for hypertension, and 16.7% for hyperglycaemia. According to our results, 27.4% of patients met one, 17.9% met two, 14.9% met three, 8.9% met four, and 3.6% met five of the MeTS criteria. 

In the multivariate analyses reported in Tables 2 and 3, the relationships between poor cognitive performance and MetS remained significant even after adjusting for other confounders potentially affecting memory (age, alcohol, education, and smoking). In the models using MetS as a continuous variable, the number of MetS criteria was associated with lower cognitive performance for verbal learning (*β* = −0.213; *P* = 0.035), short-term memory (short delay free recall: *β* = −0.256, *P* = 0.014; and short delay cued recall: *β* = −0.211, *P* = 0.048), and long-term memory (long delay free recall: *β* = −0.326, *P* = 0.002; and long delay cued recall: *β* = −0.231, *P* = 0.029). Significant associations were also found between older age, male gender, lower education levels, alcohol dependence or abuse, and memory impairment. An investigation of the individual components of MetS revealed that high triglycerides, abdominal obesity, and low HDL cholesterol were the only factors that were associated with poor memory performance. 

## 4. Discussion

This study investigated the relationships of cardiovascular risk factors with verbal learning and memory in patients with schizophrenia. Our findings provide evidence for a link between MetS and more severe memory impairment in schizophrenia. In addition, alcohol dependence or abuse also showed a significant relationship, whereas tobacco was not associated with memory impairment. These findings suggest that modifiable risk factors should be sought in patients with schizophrenia, as their optimal treatment may improve these patients' cognitive performance or keep the existing deficits from progressing.

First, we have found an association between the number of MeTS criteria present and memory impairment. Our findings confirm the link between cognitive impairments associated with MeTS in schizophrenia [3, 13], and suggest that interventions to reduce cardiovascular risk factors may have positive effects on memory deficits. Our results are also in line with previous studies' findings showing that metabolic syndrome contributes to cognitive impairment in elders [4–7]. Indeed, we found that among the different components of MeTS, hypertriglycerides, abdominal obesity, low HDL cholesterol were the most important features associated with memory impairment. These three factors are well-established risk factors for atherosclerosis [19], suggesting that atherosclerosis may be linked to cognitive impairment in schizophrenia. Several authors have also proposed nonvascular mechanisms involving actions of adiposity on neuronal tissue through neurochemical mediators, such as leptin [20–23]. Surprisingly, other factors such as hyperglycaemia and hypertension were not statistically associated with cognitive impairment in our study. These results contradict some previous studies [6, 24], specifically the study performed by Friedman et al., who found that hypertension increased the risk for cognitive impairment [13]. One explanation could be the differences between populations, especially the relatively young average age in our study (36.6 years) compared with previous studies (47.3 years in Friedman's study). Future studies are necessary to explore the influence of the length of exposure to each component of MetS on memory impairment. 

In accordance with several previous studies, alcohol dependence or abuse [25, 26] was associated with cognitive impairment. Alcohol may worsen the already decreased cholinergic transmission involved in cognitive impairment in schizophrenia [27, 28]. The neurotoxic action of alcohol, which is exerted via accelerated cerebral atrophy and reduced acetylcholine synthesis, has been described previously [29]. Interestingly, a recent study supports that alcohol use may exacerbate an existing neurobiological alteration associated with schizophrenia [30]. In practice, patients with coexisting mental and substance use disorders, such as alcohol abuse, have rarely received necessary treatments and have generally experienced poor outcomes [31, 32]. Hence, doctors should consider the possible existence of alcohol dependence or abuse when planning cognitive rehabilitation programs with these patients, and they should develop better coordination between addiction and mental health services in the treatment of these patients. 

Surprisingly, our findings did not support the existence of a relationship between smoking and memory. Nicotine is a cholinergic agonist that can counteract the cholinergic dysfunction in cognitive impairment [33]. However, previous studies found a relationship with smoking only for working memory, spatial organization, attention, and inhibitory function [28, 34]. In addition, nicotine can also desensitize/inactivate nicotinic acetylcholine receptors induced by repeated nicotine exposure [35]. Our findings should however be interpreted with caution because nicotine consumption was not precisely measured in our study (i.e., amount consumed, duration of consumption/abstinence, passive smoking).

**Limitations and Perspectives**

1. The sample may not be representative of the entire population of patients with schizophrenia. Patients were mostly middle-aged males with more than 5 years of illness duration. Thus, we need confirmation for more diverse and larger groups of patients.
2. This study is limited by being cross-sectional in design rather than prospective in design. No causal inference can be made formally between MeTS, alcohol and memory, and our models should be interpreted from an associational point of view. Future longitudinal studies are needed to confirm that the sequence MeTS/alcohol→Memory is temporally verified.
3. We only studied memory function because it is among the most commonly affected cognitive domains in patients with schizophrenia. Future studies should investigate other cognitive composites such as attention and executive function. Considering just one composite would not have been a perfect reflection of a global cognitive function. It would have been misleading to assume that our patients were not suffering from other neuropsychological deficits.
4. Several important data were not available, such as eating habits, alcohol use, and smoking details (i.e., amount and duration of consumption). Another potential weakness is related to the different medications used by patients. It appears that antipsychotics may have an impact on metabolic syndrome and cognition [36, 37]. In particular, atypical antipsychotics differ markedly in their potential to cause metabolic disturbances [38] or to improve cognition [39]. Further studies should thus investigate the complex interactions of antipsychotics, MeTS, and cognition, and examine the role of antipsychotics precisely.
5. Future studies will need to address whether preventing or lowering MeTS and alcohol use will improve cognitive impairment.
6. Finally, multiple tests were performed in our study and we cannot exclude false-positive results.

## 5. Conclusion

Our findings suggest that MetS and alcohol use may be linked to memory impairment in patients with schizophrenia. If replicated in longitudinal approaches, these findings may support novel strategies for treating and preventing cognitive disorders in patients with schizophrenia. The prevention or reduction of metabolic syndrome and alcohol use may lessen the severity of cognitive impairment in patients with schizophrenia. 

## Figures and Tables

**Table 1:** Sample characteristics (*N* = 168).

Socio-demographic characteristics	
Age in years: Mean (± SD)	36.62 ± 12.09
Sex ratio (men) (%)	73.8%
Education level (%): ≥12 y	57.6%

Clinical characteristics	

Disease duration in years: Mean (± SD)	12.15 ± 9.44
PANSS* total score: Mean (± SD)	69.45 ± 20.01
Positive factor	14.31 ± 5.45
Negative factor	19.32 ± 7.29
General psychopathology factor	35.82 ± 10.20

Second generation antipsychotics (%)	86.7%

Metabolic syndrome criteria	

Hyperglycemia (%)	16.7%
Low HDL cholesterol (%)	35.7%
High triglycerides (%)	22.6%
Hypertension (%)	39.9%
Abdominal obesity (%)	46.4%

Fagerstrom Test: Mean (± SD)	3.50 ± 3.27

Alcohol dependence or abuse (%)	27.5%

Cognitive assessment: Mean (± SD)	

Span of apprehension	
List A, trial 1	5.37 ± 2.26
Verbal learning	
Total recall Trials 1–5, List A	43.40 ± 12.85
Short-term memory	
Short delay free recall	8.67 ± 3.32
Short delay cued recall	961 ± 3.17
Long-term memory	
Long delay free recall	9.27 ± 3.60
Long delay cued recall	9.68 ± 3.43
Rate of forgetting	
Short-long-delay free recall	0.66 ± 1.51
Interference	
Proactive	−0.38 ± 2.45
Retroactive	−2.18 ± 2.26

**Table 2:** Factors associated with memory test scores: multivariate analyses (*β* ^#^).

	List A, trial 1	Total recall Trials 1–5, List A	Short delay free recall	Short delay cued recall	Long delay free recall	Long delay cued recall	Rate of forgetting	Proactive Interference	Retroactive interference
Age	−.056	−.332**	−.163	−.189	−.159	−.233*	−.028	−.154	.362**
Sex (women/men)	.100	.318**	.123	.176	.223*	.197	.289**	.073	−.318*
Educational level (<12/≥12 y)	−.052	−.338**	−.361**	−.350**	−.333**	−.328**	−.066	−.088	−.053
Number of criteria of the MetS	−.184	−.213*	−.256*	−.211*	−.326**	−.231*	−.216	.062	−.023
Fagerstrom Test	−.070	.110	.126	.167	.145	.125	.062	−.022	−.118
Alcohol dependence or abuse (yes/no)	−.010	−.038	−.123	.013	.018	.091	.279**	−.007	−.162

^#^ *β*: standardised beta coefficient (*β* represents the change of the standard deviation in neurocognition score resulting from a change of one standard deviation in the independent variable); *P ≤ 0.05; **P ≤ 0.01. MetS is included as a continuous variable in the analysis (number of MetS criteria).

**Table 3:** Individual components of MetS associated with memory test scores: multivariate analyses (*β* ^#^).

	List A, trial 1	Total recall Trials 1–5, List A	Short delay free recall	Short delay cued recall	Long delay free recall	Long delay cued recall	Rate of forgetting	Proactive interference	Retroactive interference
Abdominal obesity	−.081	−.237**	−.274*	−.276*	−.308**	−.273*	−.149	−.043	.032
Low HDL cholesterol	−.094	−.153	−.193	−132	−.211*	−.152	−.100	.069	.011
High triglycerides	−.159	−.237*	−.202*	−.195	−.240*	−.196	−.146	.056	.165
Hypertension	−.136	−.046	−.047	−.016	−.124	−.058	−.167	.110	−.104
Hyperglycaemia	−.083	.004	−.028	−.041	−.111	−.019	−.163	−.044	−.101

^#^: Adjusted for age, sex, education, smoking and alcohol use. *P ≤ 0.05; **P ≤ 0.01.
